# The instructive scar – the dynamic role of extracellular matrix in biliary pathophysiology

**DOI:** 10.1242/dmm.052835

**Published:** 2026-08-24

**Authors:** Scott H. Waddell, Euan Brennan, Luke Boulter

**Affiliations:** ^1^ Institute of Genetics and Cancer, University of Edinburgh, Crewe Road South, Edinburgh EH4 2XU, UK; ^2^ MRC Human Genetics Unit, Institute of Genetics and Cancer, University of Edinburgh, Crewe Road South, Edinburgh EH4 2XU, UK; ^3^ Cancer Research UK Scotland Centre, Institute of Genetics and Cancer, University of Edinburgh, Crewe Road South, Edinburgh EH4 2XU, UK

**Keywords:** Bile duct, Scar, Disease, Extracellular matrix, Cholangiopathies

## Abstract

Cholangiopathies – congenital and acquired diseases of the hepatic bile ducts within the liver – are often considered rare pathologies but contribute to a significant proportion of all liver transplantations. Cholangiopathies present a challenge in the clinic, often due to a lack of targeted, specific therapies, thereby underscoring the need for the development of new treatments. As with many chronic diseases, cholangiopathies exist as a spectrum; they are associated with increased inflammation and fibrosis throughout disease progression that, ultimately, result in reduced liver function and increased likelihood of developing biliary tract cancers (cholangiocarcinoma). Fibrotic scarring surrounding diseased bile ducts is characterised by aberrant changes to the molecular composition of the extracellular matrix. The contribution of individual cell types to the deposition and remodelling of the extracellular matrix, as well as its molecular composition, varies between cholangiopathies**.** Although a broad-strokes characterisation of the extracellular matrix is well established, how it conveys biochemical and biomechanical signals to the biliary epithelial cells (cholangiocytes) and how these interactions inform ductular cells to coordinate tissue-level regeneration and underpin the molecular pathology of the cholangiopathies is not well understood. In this Review, we explore the mechanisms and roles of an altered extracellular matrix in cholangiopathies and discuss the current and future perspectives for therapeutic strategies for targeting these processes in the clinic to intervene in cholangiopathies.

## Teaser

**Summary:** During chronic disease, tissue structure is disrupted by the deposition of a complex extracellular matrix. In this Review, we explore how the extracellular matrix is an active, instructive regulator of biliary development, regeneration and disease.

## An introduction to the cholangiopathies

Bile ducts are relatively simple tubular tissues made from a monolayer of biliary epithelial cells (BECs), or cholangiocytes, that form an interconnected network that span throughout the liver (from the bile canaliculi at the apical membranes of hepatocytes to the hepatic ducts at the hilum) and outside of the liver (where hepatic ducts connect to the gallbladder and duodenum via the cystic duct and common bile duct, respectively) (Raynaud et al., 2011). Cholangiopathies include congenital [e.g. Alagille syndrome (ALGS), biliary atresia (BA), polycystic liver diseases (PLDs)] and acquired [e.g. primary sclerosing cholangitis (PSC), primary biliary cholangitis (PBC), cholangiocarcinoma (CCA)] cholangiopathies, pathologies that affect the bile ducts of the liver (Fabris et al., 2019). They arise from a range of genetic, infectious, environmental and cryptic factors that result in ductular hyperplasia, abnormal morphology and the development of a surrounding fibroinflammatory niche at any position within the biliary epithelial network (Banales et al., 2019; Hirschfield et al., 2010). The result of cholangiopathies at the most advanced stages of disease is reduced biliary function (and potentially liver failure) and, in some cases, increased risk of progression to biliary tract cancer (CCA) (Banales et al., 2026).

Acquired cholangiopathies account for ∼20% of all liver transplantations in adults globally, whereas this number rises to ∼80% for congenital cholangiopathies in the paediatric population. The higher proportion in paediatric disease reflects the debilitating nature of congenital cholangiopathies (reviewed in Fabris et al., 2019), which leads to irreversible liver damage, and a higher rate of transplantation is driven by a lack of robust diagnoses and effective treatments in both acquired and inherited cholangiopathies. As a result, these complicated conditions severely reduce the quality and longevity of life for patients (Kamath et al., 2015; Lin et al., 2026; Uhlenbusch et al., 2026; Walmsley et al., 2023). Therefore, to improve patient prognosis and make disease management more accessible and sustainable, there is a need to understand the molecular mechanisms underlying cholangiopathies and develop new treatment approaches.

A common feature across cholangiopathies at the histo-molecular level is changes to the extracellular matrix (ECM) that underpin fibrosis (and/or cirrhosis) and, in the chronic setting, compromise tissue function (Hirschfield et al., 2010; Schaub et al., 2024). The mammalian ECM is composed of ∼300 proteins, and it serves not only as a structural scaffold for tissues but also as a conductor of stiffness and a reservoir to soluble factors that, upon activation, converge into intracellular biomechanical and biochemical signalling pathways to promote changes in cellular processes, such as proliferation, migration and differentiation (Bonnans et al., 2014; Saraswathibhatla et al., 2023). Remodelling of the ECM is important in normal tissue homeostasis and most commonly occurs through component degradation by matrix metalloproteinases (MMPs) and endocytosis-mediated recycling, by which ECM proteins are further processed by intracellular cathepsins. Full-length and cleaved products of the same ECM components possess different biological properties (Bonnans et al., 2014). Similarly, growth factors that sit within the matrix, such as transforming growth factor beta (TGFβ), exist in complex with latency proteins and require processing by molecular factors (including proteases and integrins) to convert them into their active form (Li et al., 2022) (Box 1). Because a wide array of regulators controls ECM homeostasis, disturbances in these pathways cause ECM dysregulation, leading to tissue pathogenesis and human disease.

Box 1. TGFβ signalling and fibrosis Transforming growth factor beta (TGFβ) is a superfamily of receptors and ligands that regulate a myriad of biological processes across development and disease (Derynck and Budi, 2019). The signalling capabilities of the TGFβ superfamily can be split into canonical (SMAD2/3-dependent) and non-canonical (SMAD2/3-independent) signalling axes, both of which are known to contribute to fibrosis (Deng et al., 2024; Kim et al., 2018) across organs, including the liver (Ortiz et al., 2021).

Canonical TGFβ signalling is initiated when TGFβ ligands (TGFβ-1/2/3) act on and form a complex with TGFβ receptors TGFβRI (ALK5) and TGFβRII, leading to phosphorylation and activation of SMAD2/3 (Deng et al., 2024). Active SMAD2/3 form complexes with co-SMAD4 and translocate to the nucleus to drive expression of target genes, which typically include extracellular matrix (ECM) genes such as collagen, laminin, fibronectin and matrix metalloproteinases (Deng et al., 2024; Kim et al., 2018; Waddell et al., 2023), as well as integrins that interact with the ECM and regulate activation of TGFβ ligands (Deng et al., 2024; Waddell et al., 2023). The ECM, in turn, regulates TGFβ signalling by sequestering latent TGFβ complexes through latent TGFβ-binding protein-1 (LTBP-1), which anchors latency-associated peptide (LAP)-bound TGFβ-1 within the matrix. Engagement of LAP by αv-containing integrins generates mechanical forces that activate and release bioactive TGFβ-1 from its latent complex (Buscemi et al., 2011; Li et al., 2022; Munger et al., 1999).

Cells detect changes to the ECM through multiple transmembrane receptors. The biggest family of these receptors are integrins, which regulate actin dynamics following ligand binding and receptor dimerisation (Hynes, 2002; Kechagia et al., 2019). On one hand, integrins allow the ECM to mediate intracellular changes, so-called outside-in signalling. On the other hand, changes in the actin cytoskeleton can regulate integrin binding affinity to ligands, so-called inside-out signalling, regulating cell adhesion and ‘pull–push’ forces exhibited by cells on a substrate (Kechagia et al., 2019). Integrins, therefore, exhibit bidirectional signalling and can regulate the signalling from the ECM to the cell and vice versa. For example, the αv-containing integrin heterodimers αvβ6 and αvβ8 are widely recognised as activating TGFβ ligands in the ECM. These integrins cleave TGFβ ligands from their latency complexes (Li et al., 2022) or interact with other TGFβ-activating molecules, such as MMP14 (Hynes, 2002) in a wide range of tissues, as well as in liver fibrosis (Schaub et al., 2024).

The role of ECM remodelling in fibrotic diseases of the liver has been reviewed previously (Ortiz et al., 2021). However, the role of and changes in the ECM, specifically, in cholangiopathies in humans have been poorly characterised and largely rely on animal models. Given the nature of chronic cholangiopathies – occurring along a spectrum, in which the disease progresses to fibrosis and subsequent cirrhosis – the evolution of the ECM composition and its temporal contribution to disease progression are not well understood. Although acquired and congenital cholangiopathies arise through distinct genetic and environmental mechanisms, many converge on profound remodelling of the ECM.

A more complete molecular understanding of cholangiopathogenesis and the identification of shared and disease-specific patterns of ECM remodelling is essential for understanding disease progression, developing targeted therapeutic strategies and determining whether individuals in an early phase of disease would benefit from the same therapeutics as those in an advanced disease state. In this Review, we first examine three congenital disorders of the biliary tree that exhibit diverse histopathological features. Despite their distinct clinical and pathological presentations, these diseases display recurring themes of ECM remodelling. We then turn to acquired fibroinflammatory cholangiopathies, which typically develop later in life, to explore whether they similarly exploit common ECM components and signalling pathways to drive pathological ductular remodelling and regeneration. Based on these data, we then address current perspectives on ECM-modulating therapeutics for the treatment of cholangiopathies.

## The role of the extracellular matrix in congenital cholangiopathies

Congenital cholangiopathies are inherited diseases and are typified by disruption to the normal morphology of the biliary network, either at the stages of its formation during embryonic development of the liver or due to structural reconfiguration of the network postnatally in the infant and continuing into adulthood (Fabris et al., 2019).

**Fig. 1.**
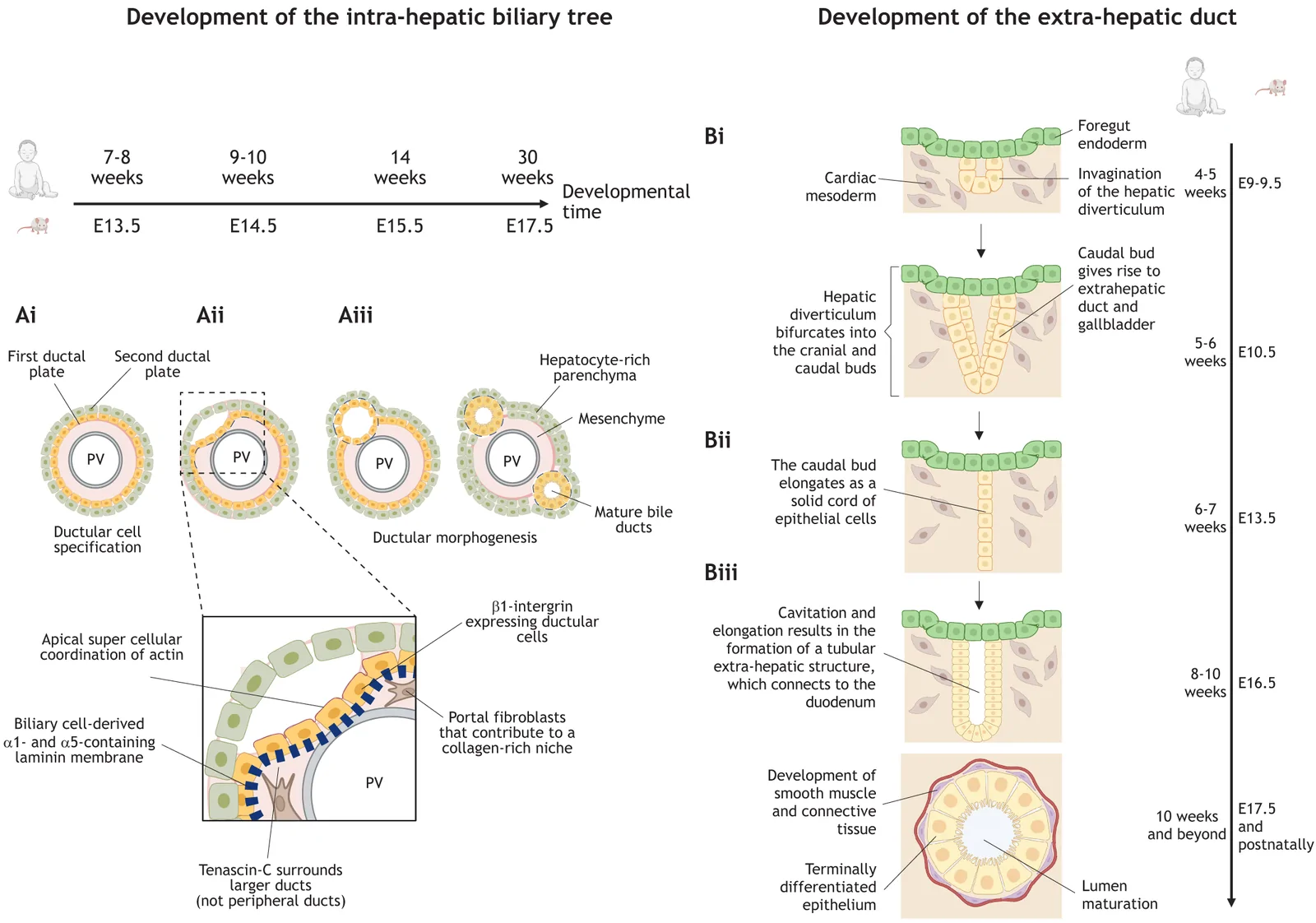
**Intra- and extra-hepatic ducts have diverse origins and compositions.** During development of the liver proper, hepatoblasts adjacent to the portal vein become the first (yellow) and second (green) ductal plates. (Ai,Aii) These ductal plates undergo morphogenesis to form a complex tubular network, in which they lumenise. This differentiation is supported by the deposition of a laminin-, collagen- and tenascin-rich extracellular matrix by mesenchymal fibroblasts adjacent to the ductal plate. (Aiii) Following lineage specification, from ∼E16.5, ducts undergo elongation, intercalation (to form a continuous biliary tree) and maturation. (Bi) During extrahepatic bile duct development, the foregut endoderm invades the cardiac mesoderm, forming the hepatic diverticulum. Following this (at E10.5 in mice and 5-6 weeks in humans), the diverticulum bifurcates to form the cranial and caudal buds, the latter of which becomes the extrahepatic duct and gallbladder (the cranial bud contributes to the liver proper). (Bii) The caudal bud of the hepatic diverticulum elongates to form a biliary cord. (Biii) The biliary cord undergoes cavitation and lumenisation concurrent with elongation where it integrates with the embryonic duodenum. Following this, the extrahepatic duct undergoes maturation and is surrounded by a smooth muscle and connective tissue layer, which supports its structure. E, embryonic day; PV, portal vein. Created in BioRender by Boulter, L. (2026). https://BioRender.com/65rusnw. This figure was sublicensed under CC-BY 4.0 terms.

The biliary tree is defined by two anatomical classifications: the intrahepatic biliary tree, which is found within the liver proper, and the extrahepatic bile duct, which resides outside of the liver, connecting it to the pancreas (via the pancreatic duct) and the duodenum. During embryonic development, BECs are specified from liver progenitor cells [hepatoblasts, which can give rise to both BECs and hepatocytes (Yang et al., 2017)] that form a simple epithelial sheet, termed the ductal plate [at approximately embryonic day (E)13.5 in mouse, Fig. 1Ai]. The ductal plate undergoes duplication and/or significant morphogenic events to give rise to both the first and second ductal plates, which dilate to form a lumen, by E14.5 (Fig. 1Aii). This discontinuous, primordial ductular system then undergoes elongation and intercalation to form a continuous intrahepatic biliary tree (between E15.5 and E17.5), although the exact mechanisms that regulate this process remain undefined (Fig. 1Aiii) (Hofmann et al., 2010; Lemaigre, 2003). Interestingly, hepatoblast-to-BEC differentiation is governed by both chemical signalling events [mediated by signals such as NOTCH and TGFβ (Raynaud et al., 2011)] and by the temporal regulation of ECM component expression. The latter regulates lineage specification and morphogenesis in the developing bile ducts: α1-containing laminins at the hepatoblast basement membrane affirm their specification to the BEC lineage, and α5-containing laminins regulate the formation of three-dimensional (3D) biliary structures in the developing murine liver via β1-integrin and regulation of actin polarisation within the primitive ductular cells (Tanimizu et al., 2012). Critically, the ability of hepatoblasts to form a lumen appears to be cell autonomous, and hepatic progenitor cells proliferating on laminin [derived from protein delta homolog 1 (DLK1)^+^ murine hepatoblasts] integrate α5-laminins into a pre-existing basement to promote their own morphogenesis. Whether this happens in humans, however, remains to be determined.

The mammalian extrahepatic ducts develop separately from the intrahepatic ducts. Rather than arising from a hepatoblast-derived ductal plate (that in humans originates from the cranial bud of the hepatic diverticulum), the extrahepatic ducts originate from the caudal bud in humans (Fig. 1Bi) or away from the hepatic bud completely in mice (Lemaigre, 2020). The caudal bud then invades the underlying cardiac mesenchyme and, ultimately, undergoes cavitation to form a lumen and elongation before forming a duct of the correct dimensions (Fig. 1Bii). The final extrahepatic duct is surrounded by a layer of smooth muscle and mesenchyme (Fig. 1Biii). Both the intrahepatic and extrahepatic bile ducts branch to interconnect with each other and form a continuous ductular network. Cholangiopathies can affect either portion of this ductular system or, indeed, both.

The ECM composition change and remodelling are crucial in 3D branching morphogenesis across both human and mouse tissues (Bonnans et al., 2014), including the mammary (Belitškin et al., 2021; Brownfield et al., 2013; Buchmann et al., 2021; Gomes et al., 2015) and salivary (Wang et al., 2021) glands. However, to date, a comprehensive characterisation of how ECM dynamics govern bile duct branching in development is lacking. In humans, type IV collagen, laminin and tenascin surround developing bile ducts (Terada et al., 1997), and congenital cholangiopathies associated with aberrant changes to the ECM implicate it in maintaining or regulating the normal morphology and physiology of the bile duct. Indeed, it is less clear whether ECM components are functional determinants of biliary ontogeny (McQuitty et al., 2020; Ozdogan and Arikan, 2023; Zeitlin et al., 2003). Recently, planar cell polarity (PCP) signalling has been shown to be essential for the developing bile duct in mice, as E18.5 mouse embryos carrying a disruptive mutation in the core PCP gene VANGL planar cell polarity protein 2 (*Vangl2*; *Vangl2^S464N^*) fail to form a complete, interconnected biliary network (Raab et al., 2024). Associated with this phenotype is a reduction in the number of active fibroblasts, which are known to remodel the ECM in homeostasis and disease (Bonnans et al., 2014) and have additionally been shown to be essential in mammary gland branching (Sumbal et al., 2024). Although there was no change in the levels of collagen in the developing bile ducts of *Vangl2^S464N^* mice, a lack of remodelling/deposition of other ECM components, such as α5-containing laminins (Tanimizu et al., 2012), could be responsible for the structural failure of the biliary network to interconnect and merits further functional exploration. In addition to laminins, other ECM components may influence the functioning of bile ducts. Previous work also identified the presence of collagen IV around both the hilar and peripheral ducts within the intrahepatic portion of the human biliary tree. However, not all proteins are universally present throughout the bile ducts. For example, tenascin can only be found around hilar ducts and is absent from peripheral ducts (Terada and Nakanuma, 1994). Further studies surveying the complete ECM profile in the developing bile duct are needed to shed light on its role in BEC specification and biliary morphogenesis.

### Alagille syndrome

ALGS is a multisystemic, paediatric disease characterised by bile duct paucity and cholestasis (Alagille et al., 1987), resulting in failed bile flow and the accumulation of toxic bile acids. Most individuals with ALGS carry a loss-of-function variant within jagged 1 (*JAG1*) [or, less commonly, a hypomorphic variant in notch receptor 2 (*NOTCH2*)], encoding core components of the NOTCH pathway (Warthen et al., 2006), which is a crucial molecular determinant of biliary specification in the developing liver (reviewed in Martinez Lyons and Boulter, 2021). In addition to a lack of bile ducts, the livers of individuals with ALGS show variability in the severity of their cholestasis, with some demonstrating slow progression to biliary cirrhosis and, consequently, end-stage liver disease (Kamath et al., 2012).

**Fig. 2.**
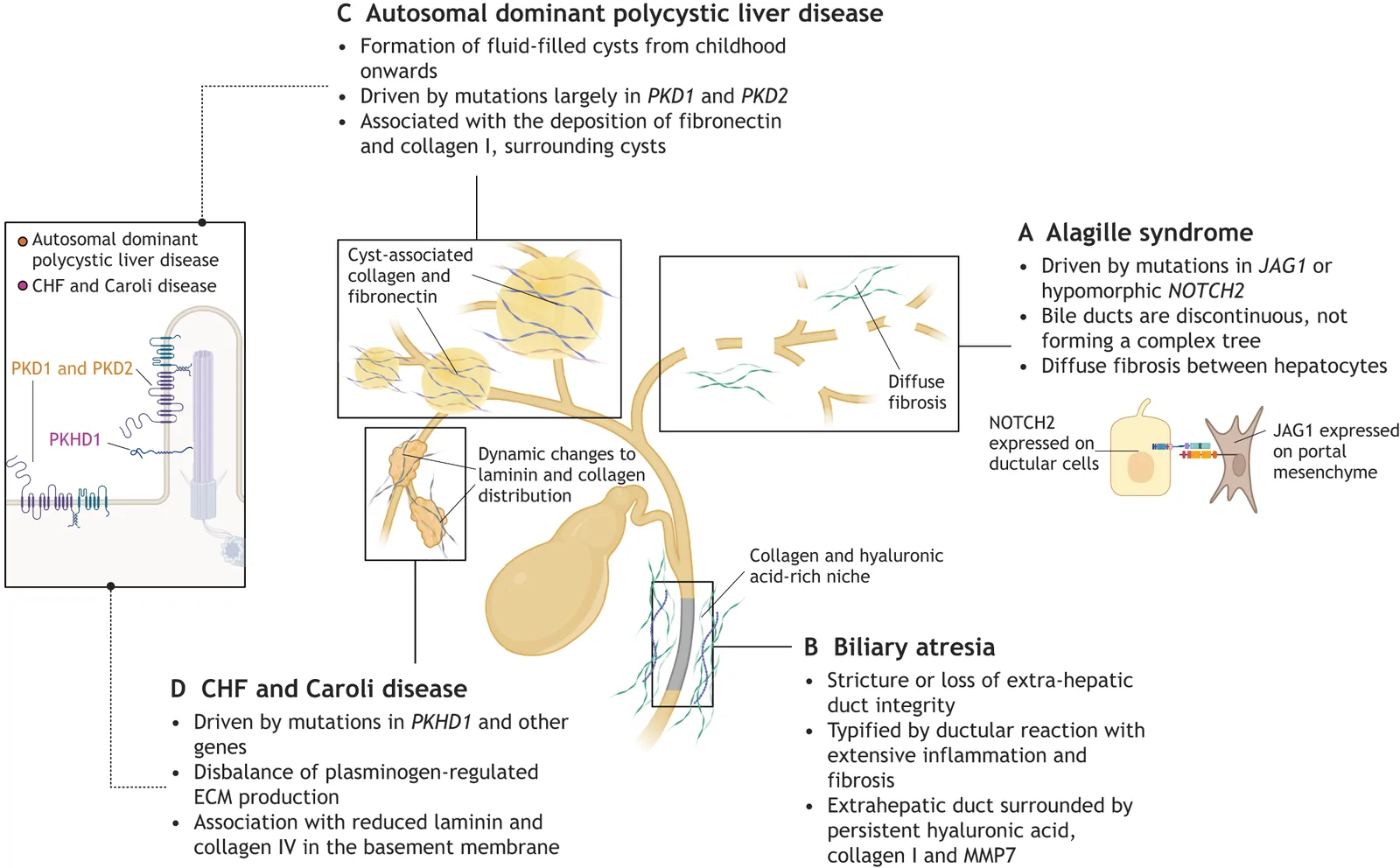
**Congenital diseases of the biliary tree.** For paediatric patients, up to 70% of liver diseases involve the bile duct. (A-D) Although a number of diverse genetic factors are known to drive Alagille syndrome (A), biliary atresia (B), polycystic liver disease (C) and congenital hepatic fibrosis/Caroli disease (D), they are all unified by progressive changes to the extracellular matrix, a common feature across developmental biliary diseases. CHF, congenital hepatic fibrosis; ECM, extracellular matrix; *JAG1*, jagged 1; MMP7, matrix metalloproteinase 7; *NOTCH2*, notch receptor 2; PKD, polycystin; PKHD1, PKHD1 ciliary IPT domain containing fibrocystin/polyductin. Created in BioRender by Boulter, L. (2026). https://BioRender.com/dixwkwn. This figure was sublicensed under CC-BY 4.0 terms.

Rather than distinct bridging fibrosis (fibrotic tracks between portal triads of the liver) and ductular reactions (a pathological feature of reactive cholangiocytes that co-evolves with a fibro-immune niche, characteristic of BA and PSC), ALGS livers present increased fibrosis between hepatocytes (Fabris et al., 2007; Mašek and Andersson, 2024), demonstrating a role for the ECM in the biliary tract pathology (Fig. 2A). In a liver-specific *Jag1* knockout mouse model (*Jag1^conditional/null^*), several ECM and ECM-modulatory genes are differentially expressed within the liver, resulting in fibrosis within the first month of life. Critically, this fibrosis resolves with age (Underkoffler et al., 2013). However, although this model demonstrates ALGS-characteristic abnormal bile duct growth in adults, it seems to demonstrate biliary hyperproliferation (rather than duct paucity) and bridging fibrosis (Loomes et al., 2007), which are not observed in individuals with ALGS. These discrepancies raise doubts about whether the *Jag1* knockout mouse model accurately reflects ALGS, but confirms Notch-mediated regulation of ECM genes (either directly or indirectly) in ALGS pathogenesis. Indeed, NOTCH has previously been described to promote fibrosis in non-cholestatic liver disease, particularly in individuals diagnosed with metabolic dysfunction-associated steatohepatitis (MASH) (Kang et al., 2023; Ramachandran et al., 2019).

In an alternative model of ALGS, in which mice harbour a germline missense mutation in the causative gene, *Jag1* (*Jag1^Ndr/Ndr^*), the clinical features of ALGS are phenocopied (Andersson et al., 2018). The observed symptoms include bile duct paucity and hepatocellular fibrosis (rather than bridging fibrosis) (Mašek et al., 2024), making this model a valuable tool for understanding how the ECM promotes the development of a normal biliary network. It may also help identify therapeutic strategies that modulate ECM remodelling and could ameliorate disease in people with ALGS.

### Biliary atresia

BA is a complex disease of the extrahepatic bile ducts (reviewed in Tam et al., 2024), affecting ∼1:5000-20,000 live births, and can lead to liver failure in early childhood (Lemaigre, 2020; Tam et al., 2024). Individuals with BA show obliteration of the extrahepatic ducts from extensive fibrosis and inflammation (Bezerra et al., 2018), with several identified factors (in patient genetics and environmental exposures) remaining loosely associated with, but not directly incriminating themselves in, BA pathogenesis (Tam et al., 2024) (Fig. 2B). Diagnosis of BA proves difficult in the clinic and is, on occasion, made late or missed entirely, thereby resulting in the development of further complications, including the progression to cirrhosis and, in some cases, to malignancy (Tam et al., 2024).

Unlike in ALGS, the livers of people with BA demonstrate classical bridging fibrosis and ductular reactions (Mašek and Andersson, 2024). As summarised previously (Tam et al., 2024), the healthy extrahepatic bile duct during the neonatal period is surrounded by ECM with a high content of hyaluronic acid, which reduces with age. Histological analyses of surgically resected fibrotic tissues around obliterated extrahepatic BA-affected bile ducts show persistently high levels of hyaluronic acid in adulthood and altered structural patterns of collagen type I (de Jong et al., 2023). Furthermore, there is an increase in the expression of MMP7 in BA-affected livers (Huang et al., 2005; Jiang et al., 2019; Lertudomphonwanit et al., 2017), highlighting a pathological role for matrix remodelling in disease. Recently, novel mouse models of BA have been generated by the liver-specific deletion of the polycystin family member, polycystic kidney disease 1 like 1 (*Pkd1l1*)*.* Mice deficient in *Pkd1l1* demonstrate increased fibrosis around portal tracts and increased expression of ECM components and remodelling genes (Hellen et al., 2023; Lim et al., 2024), which could provide useful tools to aid our understanding of how therapeutics that interfere with pathological ECM remodelling can prevent disease progression to cirrhosis.

### Polycystic liver diseases and ductal plate malformations

#### Autosomal dominant polycystic liver disease

PLDs are a group of autosomal dominant genetic diseases for which afflicted individuals present at least ten fluid-filled cysts of BEC lineage throughout the liver (Fabris et al., 2019; Perugorria et al., 2014) (Fig. 2C). PLD co-occurs with polycystic kidneys in autosomal dominant polycystic kidney disease (ADPKD; affecting ∼1 in 1000) or occurs in isolation in in autosomal dominant PLD [or isolated PLD; a much rarer disease, affecting ∼1 in 100,000]. Although related, ADPKD and isolated PLD are associated with mutations in different genes. ADPKD is caused by mutations in genes that encode polycystin proteins that localise to the primary cilium, *PKD1* (in ∼80% of cases) and *PKD2* (in ∼15% of cases); isolated PLD is characterised by mutations in genes that encode endoplasmic reticulum proteins and are thought to prevent the processing and trafficking of polycystin-1 (encoded by *PKD1*) to the primary cilium (Santos-Laso et al., 2020). The causative genetics, molecular mechanisms and current management options of PLDs have been reviewed previously (Olaizola et al., 2022). In brief, loss of polycystins, or disruption of the primary cilium itself, abolishes the ability of cholangiocytes to sense bile flow and luminal tonicity. This defect, together with widespread dysregulation of cholangiocyte-intrinsic signalling pathways, underpins the development of PLD. Supporting this concept, recent lineage-tracing studies in mice have shown that deletion of the essential ciliogenesis gene WD repeat domain 35 (*Wdr35*) induces extensive remodelling of the biliary tree, including cystic fission, thereby driving progressive cystogenesis. Despite the implication of several genes in the development of cysts, previous work has demonstrated that defective function and/or trafficking of the polycystins are the root cause of PLD (Besse et al., 2017; Fedeles et al., 2011), regardless of the mutated allele inherited.

When considering cyst development and progression at the tissue level, it is perhaps not altogether surprising that the mechanisms converting tubular structures into spherical cysts are shared. Indeed, published work has demonstrated that, in established cystic disease, the cystic BECs of multiple individuals harbouring a range of differing pathogenic mutations (in both ADPKD and isolated PLD genes) present the same molecular signatures, including active canonical TGFβ/SMAD family member (SMAD)3 signalling and α2β1-integrin expression (Waddell et al., 2023). As was mentioned above, both molecular signatures are associated with the ECM. TGFβ signalling results in increased ECM component production in chronic ductular disease (Henderson et al., 2013), and integrins represent the largest family of ECM-binding receptors, regulating essential cell functioning. Cystic BECs were found to secrete several ECM components, including fibronectin, collagens and a range of laminin molecules (Waddell et al., 2023). SMAD3 inhibition in cyst-bearing mice (driven by the BEC-specific deletion of *Wdr35*) shows reduced expression of fibronectin and a significant reduction in the size of cystic lumens, suggesting the connection between ECM composition around cystic BECs and cyst size. Alternatively, inhibition of the α2β1-integrin heterodimer in PLD animals shows a reduction in cyst number (Waddell et al., 2023), suggesting that matrix sensing is essential in the biological processes that underpin the formation of new cysts and that uncoupling cystic epithelial cells from their ECM-rich environment limits PLD progression.

#### Autosomal recessive polycystic liver disease and ductal plate malformation

**Fig. 3.**
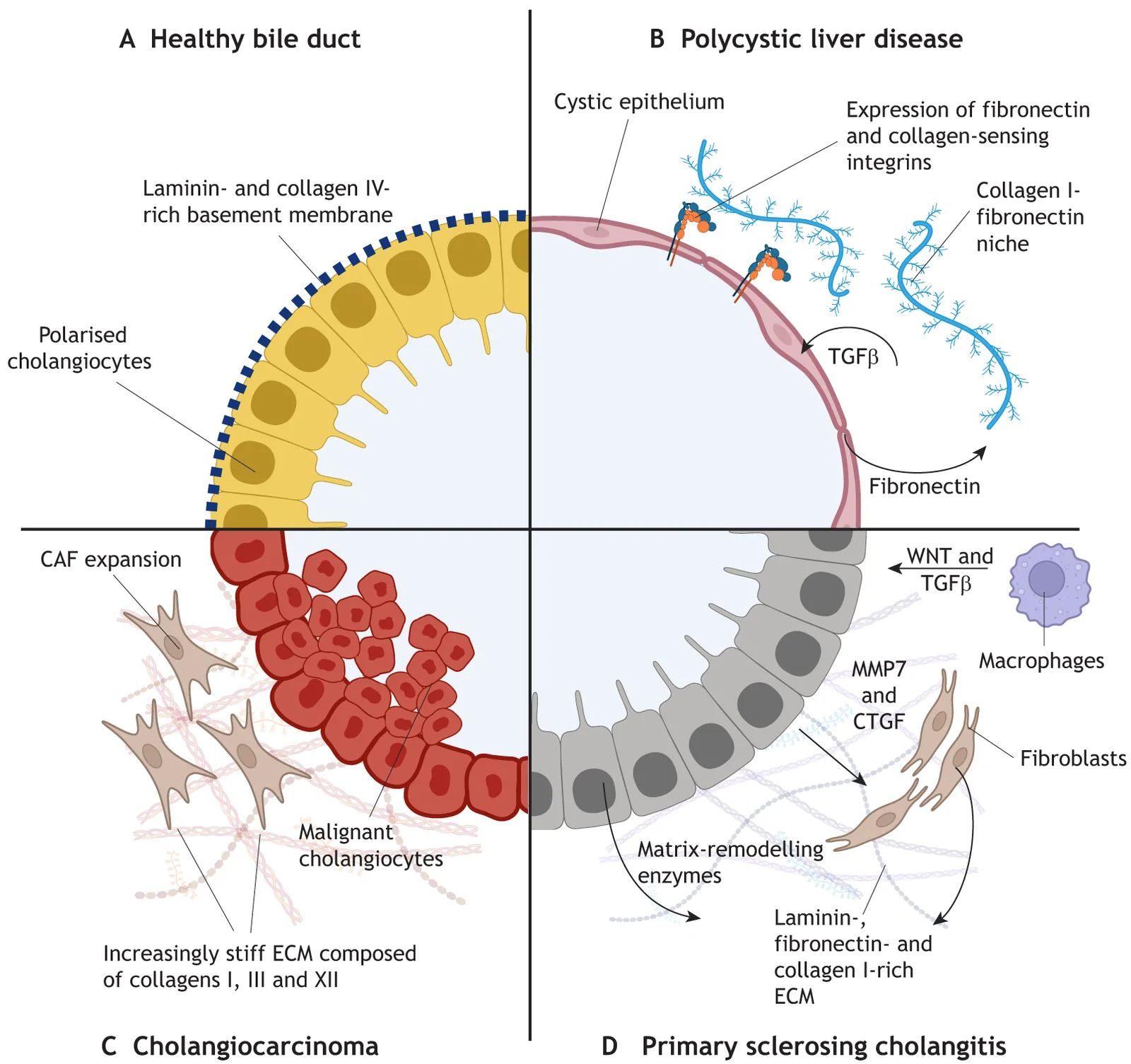
**Shared pathophysiological processes across biliary diseases.** (A) Biliary epithelial cells exist within a structured ductular network and are highly polarised, with a primary cilium characterising their apical membrane. (B-D) Throughout biliary pathologies, including polycystic liver disease (B), cholangiocarcinoma (C) and primary sclerosing cholangitis (D), biliary cells reactivate recurrent signalling cascades, deposit novel extracellular matrices and condition these scaffolds to change the structure of a tissue. Although there are common factors that influence the progression of these diseases, their histological characteristics are diverse and suggest critically different regulatory processes. CAF, cancer-associated fibroblast; CTGF, connective tissue growth factor; ECM, extracellular matrix; MMP7, matrix metalloproteinase 7; TGFβ, transforming growth factor beta. Created in BioRender by Boulter, L. (2026). https://BioRender.com/q3022go. This figure was sublicensed under CC-BY 4.0 terms.

Remodelling of the biliary tree is common in other cholangiopathies, including congenital hepatic fibrosis (CHF) and Caroli disease, which are typified by structural abnormalities in the bile ducts from birth (termed ductal plate malformations) (Gunay-Aygun, 2009) (Fig. 2D). These liver pathologies typically present in individuals with autosomal recessive polycystic kidney disease (ARPKD; presenting before early adulthood and affecting ∼1 in 20,000), caused by inheriting two mutated copies of the PKHD1 ciliary IPT domain containing fibrocystin/polyductin (*PKHD1*) gene (the protein product of which localises to the primary cilium, similarly to polycystin proteins in ADPKD). The diseased bile ducts of individuals with both CHF and Caroli disease have been shown to lack the characteristic expression of laminin and type IV collagen found within the basement membrane of BECs in health (Yasoshima et al., 2009). This loss of the stereotypical BEC basement membrane is phenocopied in a rat model of ARPKD featuring Caroli disease with CHF, known as the PCK rat. *Pkhd1-*deficient BECs derived from the PCK rat locally generate plasmin by converting plasminogen via tissue plasminogen activator, leading to laminin and collagen degradation (Yasoshima et al., 2009), as well as loss of fibronectin and connective tissue growth factor (CTGF; which interacts with fibronectin and integrins) (Fabris et al., 2022; Sato et al., 2007). This shift in ECM component abundance drives the formation of a pro-fibrotic niche. This evidence points toward a normal ECM composition promoting normal bile duct structure, whereas deviation from the biochemical and biophysical properties of the matrix promotes pathological tissue remodelling in PLD and ductal plate malformations (Fig. 3A,B).

## Aberrant ECM changes in acquired cholangiopathies

The mammalian liver is well known for its high regenerative capacity. During acute liver disease, several rodent studies have shown that hepatocyte proliferation is a major driver of tissue restoration (reviewed in Michalopoulos and Bhushan, 2021). In chronic conditions, BECs proliferate and give rise to a so-called ‘ductular reaction’, a pathophysiological response that, when hepatocyte regeneration is impaired, shows high levels of lineage plasticity and can give rise to both hepatocytes and mature BECs (Boulter et al., 2012; Martinez Lyons and Boulter, 2023; Pu et al., 2023). This population of regenerative BECs is commonly described as hepatic progenitor cells (HPCs)/liver progenitor cells (LPCs), and recent work has demonstrated a potential molecular distinction of HPCs from the majority of the BEC population (Pu et al., 2023). HPC differentiation towards different cell lineages is guided by different molecular cues (Boulter et al., 2012; Pepe-Mooney et al., 2019; Planas-Paz et al., 2019). For example, the constant presence of a laminin-rich matrix surrounding ductular reactions maintains BEC identity and inhibits their differentiation to hepatocytes (Lorenzini et al., 2010). The molecular profile of the ECM in established chronic cholestatic pathologies, however, is more complicated and is discussed below.

### Primary fibrotic/cirrhotic diseases

#### Primary sclerosing cholangitis

PSC typically presents in adulthood (Alberts et al., 2018) and is underlined by portal fibrosis (i.e. fibrosis that specifically develops around the portal triad and not the central vein within the liver) and inflammation that lead to multiple strictures of the bile ducts with potential to develop into cirrhosis and, eventually, CCA (Karlsen et al., 2017) (Fig. 3C,D). A combination of genetic and environmental factors is believed to underlie PSC aetiology. Over 20 risk genes have been associated with PSC. Most common variants are associated with the HLA gene family and antigen presentation; however, there is also evidence that genes such as macrophage stimulating 1 (*MST1*) and BCL2 like 11 (*BCL2L11*), which are involved in lymphocyte homeostasis and apoptosis, respectively, contain disease-associated variants (Karlsen et al., 2010; Melum et al., 2011; Zhou et al., 2024). In addition, up to 80% of people with PSC have co-occurring inflammatory bowel disease, which hints at a role for dysregulated gut–liver crosstalk in the PSC pathology (Karlsen et al., 2017; Munster et al., 2024; Váncza and Torok, 2023).

One key histological characteristic of the livers from individuals with PSC is the presence of concentric rings of fibrosis around bile ducts, termed onion skin scarring (Karlsen et al., 2017; O′Hara et al., 2017), composed of dense networks of structural ECM proteins, including collagen and fibronectin (Saffioti et al., 2021; Wu et al., 2016). The functional contributions of different ECM components to PSC progression are not known, largely due to a lack of robust animal models of PSC. Current animal models reflect the histopathology of PSC but lack the autoimmune profile of human disease. Multiple matrix genes have been identified as upregulated in individuals with PSC, including those that encode collagens, laminins and fibronectin (Govaere et al., 2019). Although collagens are clearly deposited histologically in PSC, there is also a concurrent increase in collagen triple helix repeat containing-1 protein (CTHRC1), which inhibits collagen synthesis relative to that in unaffected controls, suggesting that there is a dynamic collagen environment within PSC (Li et al., 2019). Indeed, the levels of fibronectin and collagen have been reported to increase in line with disease severity in affected humans and in mice fed a diet of 0.1% 5-diethoxycarbonyl-1,4-dihydrocollidine (DDC), which models cholestatic injury similar to that with PSC (Klaas et al., 2016). Given the central role of fibrogenic cells in other chronic diseases, it is postulated that hepatic stellate cells (HSCs) and portal fibroblasts exist within and contribute to the development of these layers of periductal fibrosis (Karlsen et al., 2017), but the exact contribution of each has yet to be elucidated (Box 2).

Box 2. Fibroblasts in liver health and disease The liver contains resident fibroblast populations that are activated in disease and contribute to fibrosis and pathogenesis (Kisseleva and Brenner, 2021). Hepatic stellate cells (HSCs) sit within the space of Disse, a histo-anatomical area between hepatocytes and sinusoid endothelial cells (that form the capillary network of the hepatic lobule). In health, HSCs normally rest quiescent and contribute to liver homeostasis, including functioning as the preliminary source of vitamin A (Cogliati et al., 2023; Kisseleva and Brenner, 2021). Portal fibroblasts sit around the bile ducts and portal vasculature of the liver, and are believed to contribute to the physical structure of the portal tract. However, their exact function is not fully understood (Wells, 2014).

The specific role of individual fibroblast populations to fibrosis and disease is still actively debated within the field. Despite portal fibroblasts residing closer to the biliary tree, HSCs are the major source of activated myofibroblasts in biliary injury (Mederacke et al., 2013), as well as the origin of the majority of cancer-associated fibroblasts in cholangiocarcinoma (Affo et al., 2021). Indeed, single-cell analyses of several murine models of liver injury have demonstrated that a population of portal mesenchymal stem cells expands to generate a pool of myofibroblasts that produce collagen alpha-1(XV) chain (COL15A1). These myofibroblasts promote collagen-basement membrane interactions and fibrosis, and are suspected to interact with and activate HSC-derived myofibroblasts (Lei et al., 2022). Recent data from individuals with primary sclerosing cholangitis and primary biliary cholangitis indicate that HSCs, which represent a more abundant population than portal fibroblasts, interact not just with portal fibroblasts of the fibrotic niche, but also possibly with CD4^+^ T cells (Affo et al., 2021).

Recent work has started to dissect the role of ECM in ductular remodelling in PSC. Indeed, analysis of active, ECM-producing fibroblasts [myofibroblasts; actin alpha 2, smooth muscle (*ACTA2*)*^+^*, platelet derived growth factor receptor alpha (*PDGFRA*)*^+^*, fibroblast activation protein alpha (*FAP*)*^+^* cells] from a single-cell dataset of human PSC (Andrews et al., 2024) reveals a significant increase in the numbers of putative ligand–receptor interactions of these myofibroblasts with BECs compared to those in non-diseased liver (Walker et al., 2025 preprint) (PSC tissues were collected in Canada and at point of transplant owing to decompensated cirrhosis or end-stage liver disease). This dataset shows that the main form of cell–cell communication in diseased liver is myofibroblasts producing ECM signals, sensed by BEC integrins. In healthy liver, this pattern is far less prominent. In addition to myofibroblasts, BECs have been reported to modulate their own surrounding ECM in PSC (Wilson et al., 2020). Mouse models of cholestatic injury and ductular fibrosis show that damaged BECs express collagen-modulatory enzymes, including a collagen crosslinking enzyme lysyl oxidase-like 2 (LOXL2) (Ikenaga et al., 2017), and collagen synthesis-related enzyme prolyl-4-hydroxlases (Zhang et al., 2023). These enzymes were also found to be expressed in PSC and PBC-affected liver tissues (discussed below) (Zhang et al., 2023). Both mentioned enzyme classes are important for biliary regeneration, as inhibition of either of them causes a subsequent reduction in collagen within the liver and results in limited BEC proliferation and ductular reaction (Ikenaga et al., 2017; Zhang et al., 2023).

In addition to myoblasts and BECs, immune cells and, particularly, macrophages have been reported to contribute to ECM remodelling and ductular scarring in cholestatic injury (Wilson et al., 2020). A previous report using the same mouse model of biliary injury from DDC diet showed that macrophage-derived WNT5A activates non-canonical WNT signalling in BECs, promoting the transcription of pro-fibrotic genes *Ctgf* and *Mmp7* and, therefore, scar deposition (Wilson et al., 2020). Notably, CTGF was also found to be expressed in the BECs of human cirrhotic livers (Wilson et al., 2020), suggesting that BEC-produced CTGF, which regulates fibroblast activation and collagen production is a conserved disease response across mammals. A separate study used a DDC-induced mouse model of ductular regeneration to demonstrate that αvβ6-integrin-mediated processing of TGFβ1 is downregulated in response to the genetic loss of *Ctgf*, leading to an overall reduction in hepatic fibrosis (Pi et al., 2015). Similarly, early disease intervention by pharmacological [by targeting the membrane-bound O-acyltransferase, porcupine thereby preventing WNT ligand production (Wilson et al., 2020)] or genetic (by introducing mutations into the core PCP gene, *Vangl2*) inhibition of key signalling components within the non-canonical WNT signalling axis results in reduced ECM gene expression and matrix remodelling (Wilson et al., 2020), leading to reduced fibrosis and scar deposition.

#### Primary biliary cholangitis

PBC is an autoimmune disease of the biliary tract associated with cholestasis and progressive fibrosis that can ultimately lead to end-stage biliary cirrhosis (reviewed in Gulamhusein and Hirschfield, 2020). Similar to PSC, there is a lack of understanding of the cellular and molecular mechanisms of fibrosis underlying this disease. There appear to be some similarities between PBC and PSC patient groups, including an increase in the presence of CTHRC1 (known to inhibit collagen synthesis) (Li et al., 2019). However, mechanisms of matrix regulation in PSC and PBC are different (Bauer and Habior, 2022). Although limited information exists on the differences between PSC and PBC, one study reported a specific increase in serum MMP3 levels (a protein known to degrade ECM components, as well as activate other MMP genes) in patients with PBC compared with those with PSC (Bauer and Habior, 2022).

Overall, there is a clear lack of understanding of the molecular basis for ECM modulation in primary fibrotic/cirrhotic diseases, such as PSC and PBC. Uncovering these mechanisms could help identify potential therapeutic strategies for improving patient prognosis and disease management.

### Cholangiocarcinoma

CCA is a heterogeneous group of aggressive malignancies arising from the biliary tree (reviewed in Banales et al., 2020). A major risk factor for CCA is chronic hepatic inflammation, driven by a variety of conditions, including PSC, untreated infection with the liver fluke *Opisthorchis viverrini*, other liver infections and cirrhosis (Cadamuro and Strazzabosco, 2022). Critically, chronic disease contributes to the formation of a pronounced desmoplastic reaction in intrahepatic CCA (iCCA), which forms a dense fibrotic stroma comprising of cancer-associated fibroblasts (CAFs) and ECM. The stroma in iCCA is almost four times stiffer than that in hepatocellular carcinoma. Chronic fibrosis driven by underlying chronic disease can be seen as a fibrotic niche permissive of iCCA. Indeed, the stroma of iCCA has been shown to be largely fibrotic and to contain high levels of extracellular and matricellular proteins. Furthermore, chronic stricturing in the extrahepatic portion of the biliary tree also drives the formation of chronic hepatic fibrosis (Banales et al., 2026). Increased stiffness and CAF abundance positively corelates with more aggressive disease progression across epithelial tumours (Hayashi et al., 2012; Nicolás-Boluda et al., 2020). Tumour stiffness in CCA is largely driven by the high number of CAFs, which cause chemorefractory (failure to respond to chemotherapies) and (in some cases) immune-cold nature (failure for immune cells to enter the tumour microenvironment) of iCCA tumours. Indeed, this highly desmoplastic stroma is thought to act as a complex physical barrier that prevents chemotherapeutics from reaching tumour cells (Affo et al., 2021; Banales et al., 2020; Herzog et al., 2023; Rizvi et al., 2018). iCCA stroma also directly influences tumour immunity through the production of immunoregulatory molecules [such as interleukin-6 (IL-6) and TGFβ] (Affo and Sia, 2026). CAF infiltration also correlates with increased tumour growth and worse patient outcomes (Chuaysri et al., 2009). Proteomic analysis of the iCCA-associated stroma shows a unique ECM profile characterised by increased deposition of fibrillar collagens (types I, III and XII), reduced elastic fibres and reduced proteoglycan content, ultimately resulting in increased matrix stiffness (Carpino et al., 2019). The stiffer ECM profile is unsurprising as CCA CAFs show higher levels of transcriptional enrichment for genes associated with ECM organisation and/or biosynthesis than do non-cancer-derived hepatic fibroblasts (Utispan et al., 2010). It is important to note that the ECM in CCA is not simply a consequence of a fibroblast-dense stroma; the ECM itself is also instructive of many of the hallmarks of CCA progression. Indeed, when decellularised ECM scaffolds derived from human CCA tissues are used as a substrate to culture CCA organoids, these scaffolds promote desmoplasia, further ECM deposition, epithelial–mesenchymal transition and chemoresistance (van Tienderen et al., 2023).

Modulating substrate stiffness *in vitro* through the use of decellularised or cross-linkable hydrogels is associated with increased CCA cancer cell proliferation (van Tienderen et al., 2023; Wu et al., 2026), potentially through the translocation of mechanosensitive transcription factors YAP/TAZ. However, genetic deletion of *Col1a1* in *Lrat-Cre*-positive mouse HSCs reduced *in vivo* tissue stiffness and YAP signalling but failed to suppress tumour growth (Affo et al., 2021). These results suggest that the role of ECM and tissue stiffness in CCA growth is likely to be complex and dependent on more than the presence of single fibrillar collagens.

### Targetable nodes in biliary fibrosis

The progressive accumulation of scar tissue in congenital and late-onset biliary diseases represents an attractive therapeutic candidate for therapy. Consequently, a range of therapeutic approaches has been proposed to address scar tissue formation; however, none of these strategies has yet been widely adopted in clinical practice for ductular disease. A relatively large number of anti-fibrotic compounds undergoing clinical trials rely on an indirect approach and treat the underlying cause of a disease rather than actively modulating fibrosis. For example, in the context of parenchymal fibrosis, Resmetirom has recently been approved for the treatment of MASH, an advanced and aggressive stage of metabolic dysfunction-associated steatotic liver disease (MASLD). Critically, MASLD has been identified as a substantial risk factor in the development of cholangiocarcinoma (Banales et al., 2026). However, whether the treatment-associated reduction in fibrosis (in this case, largely mediated by the correction of disordered lipid metabolism characteristic of MASH) will confer a reduction in CCA risk remains unclear. Similarly, emerging data suggest that the GLP-1 agonist, Semaglutide, is effective in reducing MASH (Sanyal et al., 2025)-associated fibrosis. However, this is likely to be driven by changes in body condition caused by weight loss and improved management of metabolic dysfunction, and thereby may reflect a reduction in underlying pathophysiology rather than a direct antifibrotic action.

A key challenge in developing antifibrotic therapies for cholangiopathies is that fibrosis is not a uniform process. Rather, the composition and function of the ECM differ substantially between diseases, suggesting that effective interventions may need to target disease-specific matrix abnormalities rather than fibrosis. The ECM changes described throughout this Review provide several examples of such opportunities.

In PLD, cystic cholangiocytes actively generate a fibronectin- and collagen-rich niche and exhibit increased α2β1-integrin expression together with SMAD3 activation (Waddell et al., 2023). These observations suggest that matrix sensing and mechanotransduction are integral components of cyst expansion rather than secondary consequences of disease. Consistent with this concept, inhibition of α2β1-integrin reduces cyst number, whereas suppression of SMAD3 signalling limits fibronectin deposition and cyst growth in experimental models (Waddell et al., 2023). Therapeutic strategies that disrupt pathological epithelial–matrix interactions may, therefore, be particularly effective in cystic biliary disorders.

In contrast, PSC is characterised by periductal collagen- and fibronectin-rich ‘onion-skin’ fibrosis (Banales et al., 2019; Hirschfield et al., 2010) and increased expression of matrix-remodelling enzymes including LOXL2 (Ikenaga et al., 2017), MMP7 (Wilson et al., 2020) and prolyl hydroxylases (Zhang et al., 2023). These findings suggest that disease progression depends not only on matrix deposition but also on matrix maturation and remodelling. Accordingly, therapies targeting collagen crosslinking, matrix organisation or TGFβ activation may be most relevant in PSC. The recent clinical evaluation of αvβ6-integrin inhibition further supports the concept that interrupting ECM-dependent activation of latent TGFβ represents a tractable therapeutic strategy in biliary fibrosis (Schaub et al., 2024).

Importantly, not all cholangiopathies are associated with excess matrix deposition. In CHF and Caroli disease, loss of laminin and collagen IV from the biliary basement membrane, together with dysregulated plasmin-mediated matrix degradation, appears to contribute to ductal plate malformation and aberrant tissue architecture (Affo and Sia, 2026; Fabris et al., 2019; Masyuk et al., 2003). These observations raise the possibility that restoring basement membrane integrity, rather than simply suppressing fibrosis, may represent a more appropriate therapeutic objective in selected developmental cholangiopathies.

Similarly, observations in BA suggest that persistent retention of a hyaluronic acid-rich matrix, together with altered collagen-I organisation and elevated MMP7 expression, may actively contribute to progressive ductal obliteration (de Jong et al., 2021; Huang et al., 2005; Jiang et al., 2019; Lertudomphonwanit et al., 2017). Such findings imply that therapies aimed at modifying matrix composition or remodelling dynamics may be more effective than strategies that broadly inhibit collagen production alone.

Finally, studies in cholangiocarcinoma suggest that the ECM functions as more than a structural barrier. The tumour-associated matrix is enriched in fibrillar collagens and displays increased stiffness, promoting mechanosensitive signalling, epithelial plasticity, chemoresistance and immune exclusion (Affo and Sia, 2026; Marin et al., 2020; Sirica and Gores, 2014; Walker et al., 2025 preprint). These findings argue that future therapies should focus not only on reducing stromal abundance but also on altering the biomechanical and signalling properties of the tumour ECM. Such approaches may improve both drug delivery and anti-tumour immune responses.

Collectively, these observations suggest that the therapeutic value of targeting the ECM will depend on understanding which matrix alterations are functionally important in a given disease state, rather than treating fibrosis as a single pathological entity. Future therapies are, therefore, likely to be most effective when matched to the dominant ECM pathology, whether this involves aberrant matrix deposition, pathological matrix remodelling, altered mechanotransduction or loss of normal basement membrane architecture. Viewed in this way, the ECM is not merely a fibrotic end-product but an instructive determinant of disease behaviour and, therefore, a source of disease-specific therapeutic vulnerabilities.
